# Versatile synthesis of the signaling peptide glorin

**DOI:** 10.3762/bjoc.13.27

**Published:** 2017-02-08

**Authors:** Robert Barnett, Daniel Raszkowski, Thomas Winckler, Pierre Stallforth

**Affiliations:** 1 Leibniz Institute for Natural Product Research and Infection Biology, Hans Knöll Institute – HKI, Junior Research Group Chemistry of Microbial Communication, Beutenbergstr. 11, D-07745 Jena, Germany; 2 Faculty of Biology and Pharmacy, Institute of Pharmacy, Department of Pharmaceutical Biology, University of Jena, Semmelweisstrasse 10, D-07743 Jena, Germany

**Keywords:** *Dictyostelium*, glorin, multicellularity, *Polysphondylium*, signaling molecules, social amoebae

## Abstract

We present a versatile synthesis of the eukaryotic signaling peptide glorin as well as glorinamide, a synthetic analog. The ability of these compounds to activate glorin-induced genes in the social amoeba *Polysphondylium pallidum* was evaluated by quantitative reverse transcription PCR, whereby both compounds showed bioactivity comparable to a glorin standard. This synthetic route will be useful in conducting detailed structure–activity relationship studies as well as in the design of chemical probes to dissect glorin-mediated signaling pathways.

## Introduction

The emergence of multicellularity from unicellular ancestors is considered a major evolutionary transition [1]. This transition has occurred not only once, in fact more than 25 independent instances of this event are known. The resulting increase in biological complexity requires fine-tuned differentiation and cell–cell communication mechanisms. The social amoebae are exquisite organisms to study the emergence of multicellularity since they can exist in both a unicellular and a multicellular stage with a well-orchestrated developmental cycle linking the two [2]. The unicellular amoebae feed on bacteria and divide by binary fission. Upon depletion of their food source, they aggregate to form a multicellular organism. Eventually, they culminate in fruiting bodies to spread some of the population as dormant spores into the environment. Secondary metabolites often constitute the key signaling molecules in these developmental processes [3–4]. For instance, aggregation of the amoebae is initiated by pulses of chemoattractive, low-molecular weight signaling molecules – so-called acrasins [5]. Additionally, it has been shown that natural products are also involved in interspecies interactions of social amoebae and bacteria [6–10]. A detailed investigation of both inter- and intraspecies interactions will give insight into the fundamentals of cell signaling and access to a rich source of novel natural products.

**Figure 1:**
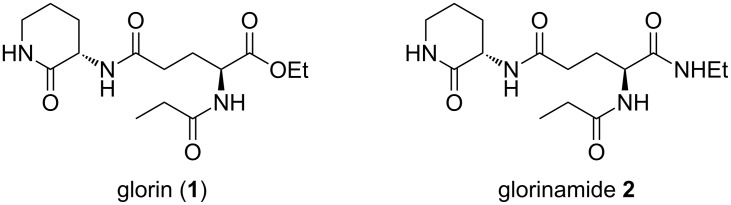
Glorin (**1**) and glorinamide **2**.

We describe a practical synthesis of the modified dipeptide glorin (**1**, Figure 1), the assumed acrasin for many of the early-diverged species of social amoebae. While numerous species of social amoebae such as *Polysphondylium pallidum, Dictyostelium fasciculatum* [11], and *D. caveatum* [12], amongst others respond chemotactically to glorin (**1**), the acrasin has only been isolated from *P. violaceum* [13]. Despite its crucial role in the initiation of multicellularity, little is known about glorin’s biosynthesis, signaling pathways, or degradation. To facilitate further studies, our chemical route allows for a facile synthesis of glorin derivatives and glorin-based chemical probes.

Here, we report the synthesis of glorin (**1**), as well as the novel synthetic analog glorinamide **2**: a compound with comparable bioactivity, that is hydrolytically – and thus metabolically – more stable than glorin (**1**) [14]. The molecules were shown to be bioactive, effectively mediating the induction of gene expression during early development, as determined by quantitative reverse-transcription PCR (RT-qPCR).

## Results and Discussion

### Synthesis of glorin and glorinamide

Two glorin syntheses have been published [15–16], one of which lacked sufficient data to be reproducible and the other one displayed limited versatility. Therefore, we focused on designing a robust synthesis that would allow for facile access to glorin derivatives required for structure–activity relationship studies. Eventually, these studies can lead to the construction of various chemical probes to identify the unknown glorin receptor.

**Scheme 1:**
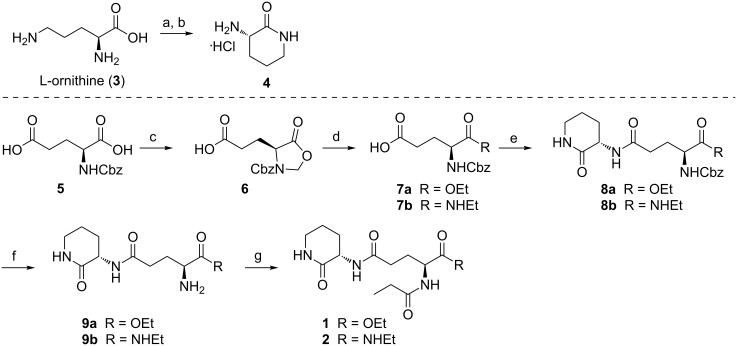
Synthesis of glorin (**1**) and glorinamide **2**. Reagents and conditions: a) TMSCl, MeOH, rt, 12 h; b) NaOEt, EtOH, 0 °C to rt, 30 min, 97% over two steps; c) H_2_CO, *p-*TsOH, toluene, Dean–Stark conditions, 3 h, 76%; d) for **7a**: NaOEt, EtOH, 0 °C to rt, 30 min, 76%, for **7b**: H_2_NEt, THF, rt, 16 h, 75%; e) for **8a**: isobutyl chloroformate, NMM, **4**, DMF, −15 °C to rt, 2 h, 69%, for **8b**: HBTU, Et_3_N, **4**, DMSO, rt, 3 h, 69%; f) Pd/C, H_2_, MeOH, rt, 1 h, 74% **9a**, 97% **9b**; g) iPr_2_EtN, DMAP, propionic anhydride, DCM, rt, 2 h, 92% **1**, 97% **2**.

Syntheses of glorin and glorinamide (Scheme 1) started from commercially available L-ornithine (**3**) and benzyloxycarbonyl-protected L-glutamic acid **5**. In an improvement on previous two-step syntheses, L-ornithine δ-lactam **4** was synthesized from L-ornithine in a one-pot procedure, whereby the latter was converted into the corresponding methyl ester using trimethylsilyl chloride in methanol [17] and cyclization was achieved under basic conditions using sodium ethoxide; the lactam was prone to racemization under strongly basic conditions, this was avoided by short reaction times with sodium ethoxide, and by avoiding strongly basic reaction conditions in subsequent steps. A key challenge in the syntheses of glorin and analogs is the differentiation between the α- and the γ**-**carboxylic acid groups of L-glutamic acid for selective esterification or amidation. α-Selective functionalization was achieved via synthesis of oxazolidinone **6** from Cbz-L-glutamic acid (**5**) with paraformaldehyde and *p*-toluenesulfonic acid under dehydrating conditions [18]. Opening of the oxazolidinone with sodium ethoxide as nucleophile thus yielded ester **7a**, while addition of ethylamine yielded amide **7b**. Subsequent amide bond formation with lactam **4** using isobutyl chloroformate or HBTU as coupling reagents furnished the protected dipeptides **8a** and **8b**, respectively. Hydrogenolysis with hydrogen gas and palladium on charcoal gave free amines **9a** and **9b**. Glorin (**1**) and glorinamide **2** were then obtained by treating amines **9a** and **9b**, respectively, with propionic anhydride. The main advantage of our synthesis over previous syntheses is that ours allows for late-stage functionalization of the α-amino group of the glutamic acid moiety. This is particularly useful for the rapid generation of different α-amide analogues.

### Biological activity of glorin and glorinamide

**Figure 2:**
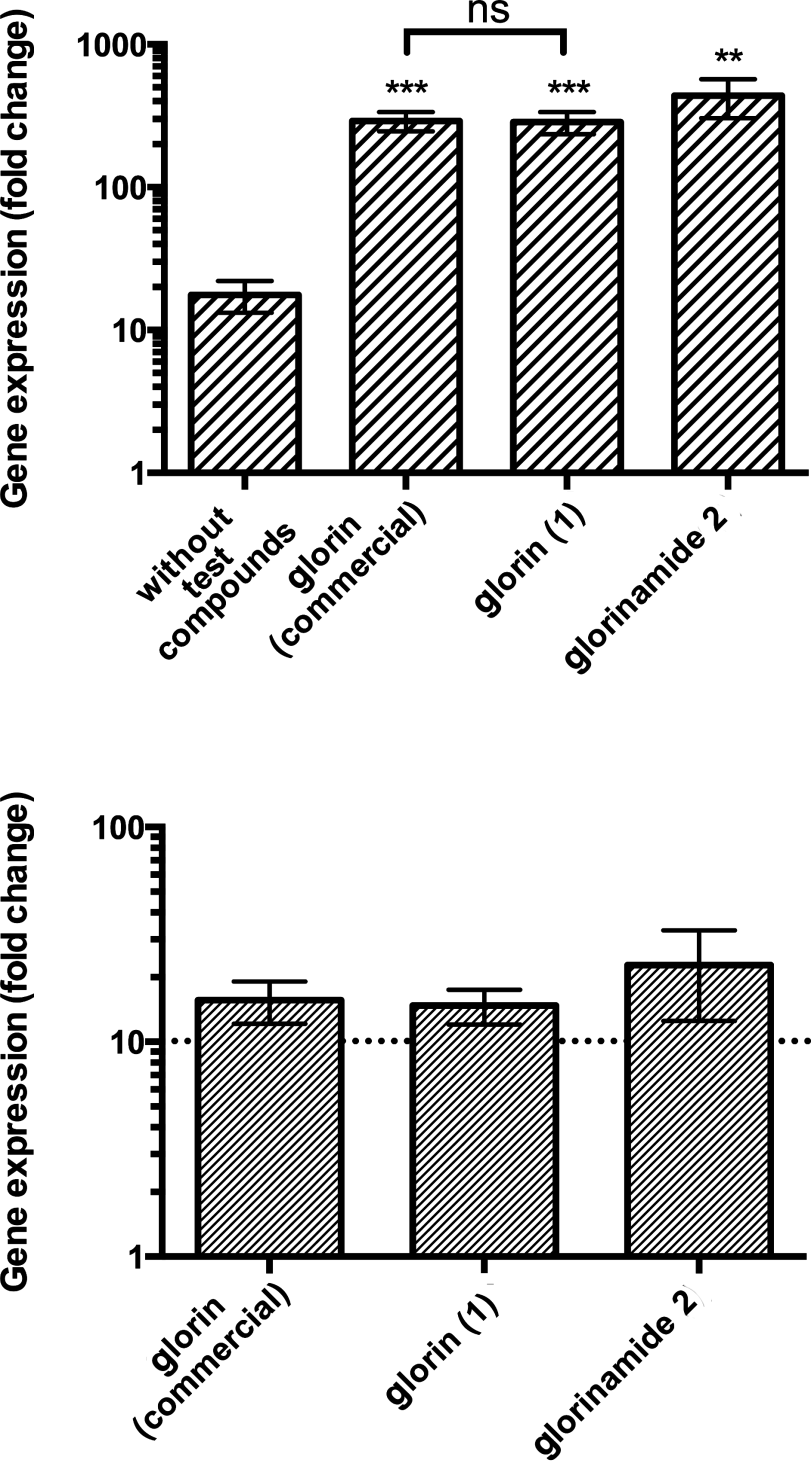
Gene induction in *P. pallidum* (model gene *PPL_09347*) without test compounds (negative control), commercial glorin (positive control), synthetic glorin (**1**) and glorinamide **2**. Top: Gene expression is presented as ‘fold change’ of gene expression in stimulated vs vegetatively-growing cells. Data are means of three biological replicates ± S.D. **: *p* < 0.01; ***: *p* < 0.001 vs vegetative cells (Student’s t-test). Bottom: Gene expression presented as ‘fold change’ of glorin-stimulated vs unstimulated cells. Data are means of five biological replicates ± S.D. and GraphPad Prism 6 was used for visualization of the results.

The bioactivities of synthetic glorin (**1**) and glorinamide **2** were assayed by their ability to elevate the expression of the glorin-induced gene *PPL_09347* in the social amoeba *P. pallidum,* as previously determined [11]. We chose the gene *PPL_09347*, which encodes the *Dictyostelium discoideum* ortholog of profilin I, an actin binding protein required for the actin cytoskeleton organization, as a model gene. Upon glorin (**1**) exposure, this gene was found to be up-regulated by about 50-fold [11]. To this end, the compounds were dissolved in DMSO/water and added in two 1 μM portions (30 min apart) to a suspension of starving *P. pallidum*. After 1 h, the cells were harvested and total RNA was extracted. The differential regulation of *PPL_09347* was determined by RT-qPCR. Synthetic glorin (**1**) and glorinamide **2** led to similar expression of *PPL_09347*, comparable to commercial glorin as positive control (Figure 2). While a small baseline induction of *PPL_09347* without test compounds is always observed (Figure 2, top), induction by glorin and glorinamide led to a significant increase in expression level above baseline (Figure 2, bottom).

## Conclusion

In summary, we have devised a versatile and robust synthetic route to glorin that allows for a wide range of derivatizations. Glorin, as well as the hydrolytically more stable derivative glorinamide, were shown to display comparable glorin-induced gene expression in *Polysphondylium pallidum*. In future this synthesis will facilitate the construction of a library of glorin derivatives for a detailed structure–activity relationship study. Ultimately, we wish to synthesize chemical probes of glorin for the identification of the unknown glorin receptor.

## Experimental

**Quantitative reverse transcription PCR:*** P. pallidum* PN500 cells were cultured in association with *Escherichia coli* K12 cells. Cells were harvested before first signs of aggregation became visible. Cells were washed three times in 17 mM phosphate buffer (pH 6.2) to remove bacteria and suspended at 2 × 10^7^ cells/mL in 17 mM phosphate buffer (pH 6.2) with shaking at 150 rpm. After 1 h of starvation, 1 µM glorin (Phoenix Pharmaceuticals, Burlingame CA, USA), synthetic glorin (**1**), or glorinamide **2** (100 µM stock solutions with 3% DMSO) or water were added to the cells every 30 min for 1 h. Cells were centrifuged for 30 min after the last addition and stored in pellets of 2 × 10^7^ cells at −80 °C.

Total RNA was prepared using the QIAGEN RNeasy kit and cDNA was synthesized using the QIAGEN Omniscript kit and an oligo(dT) primer. Expression of the glorin-induced model gene *PPL_09347* was determined by RT-qPCR as described [11,19]. Expression of *PPL_09347* in different cDNA samples was standardized to the reference gene glyceraldehyde-3-phosphate dehydrogenase (*gpdA*, SACGB accession number *PPL_12017*; http://sacgb.fli-leibniz.de/cgi/index.pl).

## Supporting Information

File 1 Detailed experimental procedures, compound characterization data, and copies of NMR spectra.
