# Supplementary material for: Versatile synthesis of the signaling peptide glorin
Source: Beilstein J Org Chem. 2017 Feb 8;13:247–50. doi: 10.3762/bjoc.13.27 (PMC5331329; doi:10.3762/bjoc.13.27)

Supporting Information

for

Versatile synthesis of the signaling peptide glorin

Robert Barnett[1], Daniel Raszkowski[2], Thomas Winckler[2] and Pierre Stallforth[*1]

Address: [1]Leibniz Institute for Natural Product Research and Infection Biology, Hans Knöll Institute – HKI, Junior Research Group Chemistry of Microbial Communication, Beutenbergstr. 11, D-07745 Jena, Germany and [2]School of Biology and Pharmacy, Institute of Pharmacy, Department of Pharmaceutical Biology, University of Jena, Semmelweisstrasse 10, D-07743 Jena, Germany

Email: Pierre Stallforth - pierre.stallforth@leibniz-hki.de

*Corresponding author

Detailed experimental procedures, compound characterization data, and copies of NMR spectra

Table of contents:

Experimental section – synthesis S2

References S11

NMR spectra S12

General information

All 1D (^{1}H, ^{13}C) and 2D NMR spectra (^{1}H-^{1}H COSY, HSQC, HMBC, NOESY) were recorded in deuterated solvents (Carl Roth, Germany) on Bruker AVANCE II 300, AVANCE III 500 and 600 MHz (equipped with a Bruker Cryo Platform) instruments. The chemical shifts are reported in parts per million (δ) relative to the resonance of the residual solvent (δ_{CHCl3} = 7.24/77.0, δ_{DMSO} = 2.49/39.51, δMeOH = 3.31 / 49.0, $\delta_{pyridine}$ = 8.74/150.35 for ^{1}H and ^{13}C spectra, respectively). Coupling constants (*J*) are reported in Hertz (Hz). HRESI–MS measurements were conducted on a Thermo Fisher Exactive Orbitrap either by direct injection or in combination with a Thermo Accela HPLC system. The system is equipped with an electrospray ion source and a Betasil 100-3 C18 column (150 × 2.1 mm). The following elution gradient was used: solvent A: H_2O + 0.1% HCOOH, solvent B: acetonitrile, gradient: 5% B for 1 min, 5% to 98% B in 15 min, 98% B for 15 min, flow rate: 0.2 mL min^{-1}, injection: 5 µL. Optical rotation measurements were performed using a 0.5 dm cuvette on a JASCO P-1020 polarimeter at 25 °C (unless otherwise noted). All reagents used were reagent grade and used as supplied except where noted (purchased from Sigma-Aldrich, TCI Chemicals, or Carl Roth). Reactions were performed under ambient atmosphere except where noted. Analytical thin-layer chromatography was performed on silica gel 60 F_{254} plates (0.25 mm, E. Merck). Compounds were visualized by dipping the plates in a cerium sulfate ammonium molybdate (CAM) solution or a ninhydrin/acetic acid solution followed by heating. Liquid chromatography was performed using forced flow of the indicated solvent on silica gel 40–63 µm (Normasil 60, VWR). Hypersep C18 SPE (1 g) columns were purchased from ThermoScientific and conditioned as described.

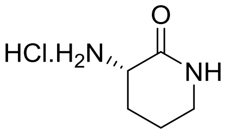

<u>(*S*)-3-Aminopiperidin-2-one hydrochloride (**4**)</u>

Trimethylchlorosilane (2.8 mL, 23 mmol, 4 equiv) was added to L-ornithine·HCl (1.0 g, 6.0 mmol, 1 equiv) followed by the addition of anhydrous methanol (20 mL). The mixture stirred at rt for 12 h. The solution was then cooled to 0 °C and a 21% (w/w) solution of sodium ethoxide in ethanol (42 mmol, 17 mL) was added; after 5 min the solution was allowed to warm to rt and stirred for another 30 min. The solution was neutralized to pH 7 with 6 N aq HCl. The resulting solution was filtered and conc. in vacuo. Salts were removed by dissolution in isopropanol, filtered, and conc. in vacuo. The crude residue was purified by flash column chromatography on silica gel (30% methanol in dichloromethane) to afford lactam hydrochloride **4** as a hygroscopic, pale yellow solid. (880 mg, 5.8 mmol, 97%). R_f = 0.2 (50% methanol in dichloromethane). ^{1}H-NMR (600 MHz, methanol-d_4): δ = 3.58-3.54 (m, 1 H), 3.32-3.27 (m, 2 H,), 2.24-2.18 (m, 1 H), 1.99-1.94 (m, 1 H), 1.91-1.82 (m, 1 H), 1.75-1.67 (m, 1 H); ^{13}C-NMR (150 MHz, methanol-d_4): δ = 172.8, 51.4, 42.8, 28.4, 21.9 ppm. $[\alpha]_D^{25}$ = 7.0 (c 0.5, methanol); HRMS (ESI+): calcd. for $C_5H_{11}N_2O$ [M + H$^+$]: 115.0866; found 115.0866. Spectroscopic data were in agreement with previously reported data.[1]

Marfey's derivatization of lactam **4**:

A solution of lactam **4** (1 mg, 7.0 μmol, 1 equiv), N_α-(2,4-dinitro-5-fluorophenyl)-L-alaninamide (2.2 mg, 8.0 μmol, 1.2 equiv), and diisopropylethylamine (3.7 mg, 28 μmol, 4 equiv) in dimethyl sulfoxide (1 mL) was heated for 1 h at 40 °C. The reaction mixture was then subjected to LC–MS analysis and compared to a racemic standard. The enantiomeric excess of the lactam **4** was determined to be >99%.

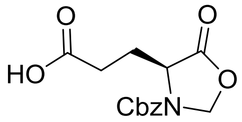

(*S*)-3-(3-((Benzyloxy)carbonyl)-5-oxo-oxazolidin-4-yl)propanoic acid (**6**)

A suspension of Z-L-Glu-OH (**5**, 1.0 g, 3.6 mmol, 1 equiv), paraformaldehyde (162 mg, 5.4 mmol, 1.5 equiv) and *p*-TsOH (10 mg, 1% w/w) in toluene (50 mL) was refluxed using a Dean–Stark apparatus for 3 h, and then allowed to cool to rt. The reaction mixture was then filtered through a silica plug equilibrated with toluene; the product was eluted with ethyl acetate, conc. in vacuo and residual solvent co-evaporated with dichloromethane to afford **6** as a colorless oil (0.80 g, 2.7 mmol, 76%). R_f = 0.2 (50% hexanes in ethyl acetate). ^{1}H-NMR (500 MHz, $CDCl_3$): δ = 7.37-7.30 (m, 5 H, phenyl), 5.49 (br s, 1 H), 5.21 (d, 1 H, *J* = 4.3), 5.17 (s, 2 H), 4.38 (t, 1 H, *J* = 5.6), 2.49-2.39 (m, 2 H), 2.31-2.23 (m, 1 H), 2.18-2.11 (m, 1 H); ^{13}C-NMR (125 MHz, $CDCl_3$): δ = 177.6, 171.6, 153.1, 135.1, 128.7, 128.7, 128.4, 77.8, 68.2, 53.9, 29.1, 25.7 ppm; $[\alpha]_D^{25}$ = +72.9 (c 0.5, methanol); HRMS (ESI+): calcd. for $C_{14}H_{16}NO_6$ [M + H^+]: 294.0972; found 294.0973. Spectroscopic data were in agreement with previously reported literature.[2]

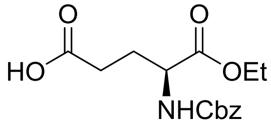

(*S*)-4-(((Benzyloxy)carbonyl)amino)-5-ethoxy-5-oxopentanoic acid (**7a**)

Acid **6** (400 mg, 1.4 mmol, 1 equiv) dissolved in ethanol (20 mL) was added to a solution of sodium ethoxide (370 mg, 5.5 mmol, 4 equiv) in ethanol (20 mL) at 0 °C. The reaction was then allowed to warm to rt and stirred for 30 min. Subsequently, 1 N aq HCl (30 mL) was added, extracted with ethyl acetate, washed with brine, dried over Na_2SO_4 and conc. in vacuo. The crude mixture was purified by flash column chromatography on silica gel (50% hexane in ethyl acetate) to afford ester **7a** as a pale yellow oil (260 mg, 0.84 mmol, 76%). R_f = 0.4 (50% hexanes in ethyl acetate). ^{1}H-NMR (500 MHz, $CDCl_3$): δ = 7.35-7.27 (m, 5 H,), 5.47 (d, 1 H, *J* = 7.7), 5.07 (m, 2 H), 4.42-4.37 (m, 1 H), 4.21-4.15 (m, 2 H), 2.48-2.38 (m, 2 H), 2.22-2.14 (m, 1 H), 1.98-1.90 (m, 1 H), 1.23 (t, 3 H, *J* =

6.9). ^{13}C-NMR (125 MHz, $CDCl_3$): δ = 177.6, 172.0, 156.1, 136.2, 128.5, 128.2, 128.1, 67.1, 61.8, 53.2, 29.9, 27.6, 14.1 ppm; $[\alpha]_D^{25}$ = –15.8 (c 0.5, methanol); HRMS (ESI+): calcd. for $C_{15}H_{19}NO_6$ [M + H^+]: 310.1285; found 310.1286. Spectroscopic data were in agreement with previously reported literature. Spectroscopic data were in agreement with previously reported literature.[3]

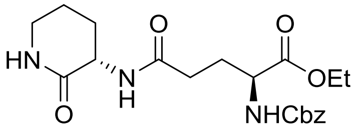

Ethyl *N*^{2}-((benzyloxy)carbonyl)-*N*^{5}-((*S*)-2-oxopiperidin-3-yl)-L-glutaminate (**8a**)

Acid **7a** (0.10 g, 0.32 mmol, 1 equiv) was dissolved in dimethylformamide (3 mL) and cooled to −15 °C, *N*-methylmorpholine (56 mg, 0.48 mmol, 1.5 equiv) in dimethylformamide (3 mL) was added, followed by isobutylchloroformate (57 mg, 0.41 mmol, 1.3 equiv). The reaction was stirred for 10 min, followed by the addition of lactam **4** (0.15 g, 0.96 mmol, 3 equiv) in methanol (1 mL). The mixture was stirred for 15 min, allowed to warm to rt and stirred for another 2 h. The crude was purified by flash column chromatography on silica gel (5% methanol in dichloromethane) to afford amide **8a** as a clear solid (90 mg, 0.22 mmol, 69%). R_f = 0.29 (5% methanol in dichloromethane). ^{1}H-NMR (300 MHz, $CDCl_3$): δ = 7.35-7.26 (m, 5 H), 6.81-6.75 (m, 1 H), 5.53 (d, 1 H, *J* = 7.8), 5.07 (s, 2 H), 4.40-4.29 (m, 1 H), 4.27-4.20 (m, 1 H), 4.20-4.12 (m, 2 H), 3.30-3.22 (m, 1 H), 2.47-2.34 (m, 2 H), 2.32-2.24 (m, 1 H), 2.22-2.13 (m, 1 H), 1.99-1.90 (m, 1 H), 1.87-1.81 (m, 1 H), 1.25 (t, 3 H, *J* = 6.8 Hz); ^{13}C-NMR (75 MHz, $CDCl_3$): δ = 176.6, 172.4, 171.9, 136.1, 128.5, 128.5, 128.2, 128.1, 67.1, 61.7, 53.3, 50.3, 41.6, 29.9, 27.6, 26.9, 20.9, 14.1 ppm; $[\alpha]_D^{25}$ = +4.9 (c 0.5, chloroform); HRMS : calcd. for $C_{20}H_{28}N_3O_6$ [M + H^+]: 406.1973; found 406.1972.

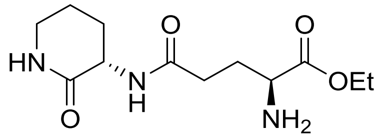

Ethyl N^5-((*S*)-2-oxopiperidin-3-yl)-L-glutaminate (**9a**)

A solution of carbamate **8a** (30 mg, 74 µmol, 1 equiv) in methanol (5 mL) was flushed with argon; Pd/C (3 mg, 10% w/w) was added, followed by bubbling H_2 gas through the solution for a few min. The solution was then stirred for 1 h under back pressure of H_2. To the reaction mixture was added methanol-washed celite and stirred for a few min.; the mixture was then filtered through a celite plug, washed with hot methanol, and conc. in vacuo to afford amine **9a** as an white solid, which was used without further purification (15 mg, 55 µmol, 74%). R_f = 0.15 (5% methanol in dichloromethane). ^{1}H-NMR (500 MHz, $CDCl_3$): δ 7.15 (d, 1 H, *J* = 6.0), 6.71 (s, 1 H), 4.34-4.29 and 4.27-4.21 (m, 1 H), 4.13-4.07 (m, 2 H), 3.42-3.38 (m, 1 H), 3.28-3.23 (m, 2 H), 2.40-2.26 (m, 3 H), 2.10-2.03 (m, 1 H), 1.89-1.80 (m, 2 H), 1.79-1.69 (m, 1 H), 1.57-1.47 (m, 1 H), 1.20 (t, 3 H, *J* = 7.1); ^{13}C-NMR (125 MHz, $CDCl_3$) δ 175.6, 172.6, 171.8, 60.9, 53.7, 50.2, 41.6, 32.4, 30.1, 27.3, 21.0, 14.1; $[\alpha]_D^{25}$ = –12.5 (c 0.2, methanol); HRMS (ESI+): calcd. for $C_{12}H_{22}N_3O_4$ [M + H^+]: 272.1605; found 272.1601.

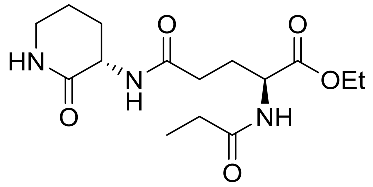

Ethyl N^5-((*S*)-2-oxopiperidin-3-yl)-N^2-propionyl-L-glutaminate, glorin (**1**)

A mixture of amine **9a** (13 mg, 48 µmol, 1 equiv), propionic anhydride (62 mg, 0.48 mmol, 10 equiv), dimethylaminopyridine (1 mg, 10% w/w), and diisopropylethylamine (60 mg, 0.48 mmol, 10 equiv) in dichloromethane (5 mL) was stirred at rt for 2 h. The mixture was conc. in vacuo, and purified by flash column chromatography on silica gel (5% methanol in dichloromethane) to afford glorin **1** as a white solid. (14.5 mg, 44 µmol, 92%). R_f = 0.45 (5% methanol in dichloromethane). ^{1}H-NMR (300 MHz, $CDCl_3$): δ 7.01

(d, 1 H, *J* = 5.8), 6.73 (d, 1 H, *J* = 7.5), 6.27 (br s, 1 H), 4.56-4.52 (m, 1 H), 4.32-4.28 (m, 1 H), 4.18 (q, 2 H, *J* = 7.1), 3.36-3.31 (m, 2H), 2.48-2.43 (m, 1 H), 2.37-2.29 (m, 2 H), 2.25 (q, 2 H, *J* = 7.6), 2.26-2.16 (m, 1 H), 2.00-1.88 (m, 2 H), 1.64-1.57 (m, 1 H), 1.26 (t, 3 H, *J* = 7.1 Hz), 1.14 (t, 3 H, *J* = 7.6 Hz); ^{13}C-NMR (75 MHz, $CDCl_3$) δ 174.2, 172.4, 172.2, 171.7, 61.4, 51.8, 50.3, 41.7, 32.2, 29.3, 27.9, 27.3, 21.0, 14.1, 9.6; $[\alpha]_D^{22}$ = +37.7° (c 0.4, chloroform); HRMS : calcd. for $C_{15}H_{26}N_3O_5$ [M + H$^+$]: 328.1867; found 328.1867.

Comparision of synthetic glorin with a previously described specimen[4]

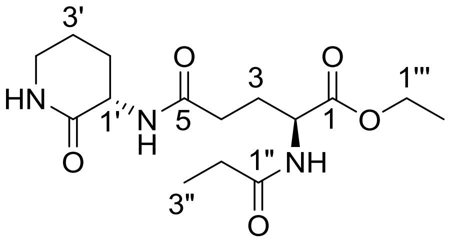

Position	δH (glorin[4] $CDCl_3$ 400 MHz)	δH (synthetic glorin $CDCl_3$ 300 MHz)
N^5	6.88, 6.80	7.01
N^1	6.76, 6.62	6.73
$N^{4'}$	6.36	6.27
2	4.67–4.61, 4.60–4.54	4.56–4.52
1’	4.40–4.33, 4.31–4.23	4.32–4.28
1’’’	4.20	4.18
4’	3.36	3.36–3.31
2’	2.50–2.37	2.48–2.43
4	2.34	2.37–2.29
2’’’	2.27, 2.26	2.25
3	2.25–2.15	2.26–2.16
3 and 3’	2.05–1.85	2.00–1.88
2’	1.72–1.58	1.64–1.57
2’’’	1.27	1.26
3’’	1.16	1.14

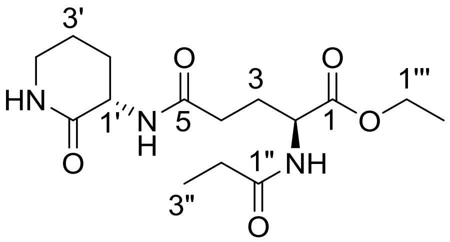

Position	δC (glorin[4] $CDCl_3$ 400 MHz)	δC (synthetic glorin $CDCl_3$ 75 MHz)
CO	174.4	174.2
CO	172.6	172.4
CO	172.2	172.2
CO	171.9	171.7
1’’’	61.5	61.4
2	51.9, 51.8	51.8
1’	50.3, 50.2	50.3
4’	41.8	41.7
4	32.4, 32.3	32.2
2’’	29.3	29.3
3	28.1, 28.0	27.9
2’	27.4, 27.4	27.3
3’	21.1	21.0
2’’’	14.1	14.0
3’’	9.7	9.6

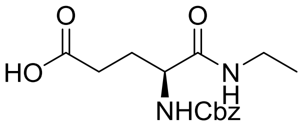

(*S*)-4-(((Benzyloxy)carbonyl)amino)-5-(ethylamino)-5-oxopentanoic acid (**7b**)

Ethylamine (2 M in tetrahydrofuran, 1 mL, 2.0 mmol, 2 equiv) was added to a solution of acid **6** (300 mg, 1.0 mmol, 1 equiv) dissolved in tetrahydrofuran and stirred at rt for 16 h. The solution was acidified with 1 N aq HCl (aq), extracted with ethyl acetate, washed

with brine, dried over Na_2SO_4 and conc. in vacuo. The crude was purified by flash column chromatography on silica gel (5% methanol in dichloromethane) to afford amide **7b** as a white solid. (236 mg, 0.77 mmol, 75%). R_f = 0.17 (5% methanol in dichloromethane). ^{1}H-NMR (500 MHz, DMSO-d_6): δ = 7.85 (t, 1 H, *J* = 5.5), 7.38-7.31 (m, 5 H), 7.317.27 (1 H, m), 5.05-4.97 (m, 2 H), 3.95-3.89 (m, 1 H), 3.11-3.00 (m, 2 H), 2.25-2.19 (m, 2 H), 1.90-1.80 (m, 1 H), 1.75-1.66 (m, 1 H), 0.99 (t, 1.8 H, *J* = 14.5). ^{13}C-NMR (125 MHz, DMSO-d_6): δ = 173.8, 170.9, 155.8, 137.0, 128.3, 127.7, 127.6, 65.3, 54.0, 33.3, 30.2, 27.3, 14.6 ppm; $[\alpha]_D^{25}$ = –14.10 (c = 0.5, MeOH); HRMS (ESI+): calcd. for $C_{15}H_{21}N_2O_5$ [M + H^+]: 309.1445; found 309.1442.

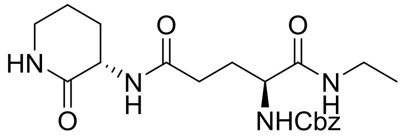

Benzyl-((*S*)-1-(ethylamino)-1,5-dioxo-5-(((*S*)-2-oxopiperidin-3-yl)amino)pentan-2-yl)carbamate (**8b**)

HBTU (2-(1*H*-benzotriazol-1-yl)-1,1,3,3-tetramethyluronium hexafluorophosphate) (74 mg, 0.20 mmol, 3 equiv) was added to a solution of acid **7b** (20 mg, 0.07 mmol, 1 equiv), triethylamine (27 µL, 0.20 mmol, 3 equiv), lactam **4** (30 mg, 0.2 mmol, 3 equiv) in dimethylsulfoxide (4 mL). The reaction was stirred at rt for 3 h, and diluted with H_2O (36 mL). A Hypersep C18 column (1 g) was washed with methanol (10 mL) and then equilibrated with 10% methanol in H_2O (10 mL). Removal of dimethylsulfoxide was achieved by loading the reaction onto the column, washing with 10% methanol in H_2O, followed by elution of the product with 50% methanol in H_2O, and conc. in vacuo. To remove HBTU by-products the crude was re-dissolved in dichloromethane, washed with H_2O, brine, dried over Na_2SO_4, and conc. in vacuo to afford **8b** as a white solid (18 mg, 0.05 mmol, 69%). R_f = 0.15 (5% methanol in dichloromethane). ^{1}H-NMR (500 MHz, DMSO-d_6): δ = 8.00 (d, 1 H, *J* = 8.1), 7.85 (t, 1 H, *J* = 5.6), 7.58 (br s, 1 H), 7.35 (m, 5 H), 7.32 (m, 1 H), 5.00 (m, 2 H), 4.18-4.12 (m, 1 H), 3.94-3.86 (m, 2 H), 3.14-3.05 (m, 2 H), 3.06 (m, 2 H), 2.20-2.08 (m, 2 H), 1.97-1.90 (m, 1 H), 1.89-1.81 (m, 1 H), 1.81-1.68 (m, 3 H), 1.61-1.52 (m, 1 H), 1.00 (t, 3 H, *J* = 7.2). ^{13}C-NMR (125 MHz, DMSO-d_6): δ =

171.6, 171.6, 170.4, 156.3, 137.5, 128.8, 128.2, 128.2, 65.9, 54.8, 49.3, 41.4, 33.9, 32.4, 28.6, 28.1, 21.5, 15.1 ppm; $[\alpha]_D^{25}$ = +7.6 (c 0.2, methanol); HRMS (ESI+): calcd. for $C_{20}H_{29}N_4O_5$ [M + H$^+$]: 405.2132; found 405.2126.

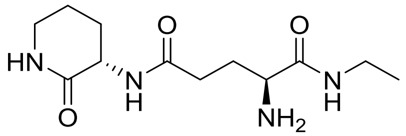

(*S*)-2-Amino-*N*[1]-ethyl-*N*[5]-((*S*)-2-oxopiperidin-3-yl)pentanediamide (**9b**)

A solution of carbamate **8b** (13 mg, 30 µmol, 1 equiv) in methanol (2 mL) was flushed with argon, Pd/C (1 mg, 10% w/w) was added, H_2 gas was bubbled through the solution for a few min. and then stirred for 1 h under back pressure of H_2. To the reaction mixture was added methanol-washed celite and stirred for a few minutes; the mixture was then filtered through a celite plug, washed with methanol, and conc. in vacuo to afford amine **9b** as a white solid, which was used without further purification (9 mg, 0.03 mmol, 97%). R_f = 0.1 (20% methanol in dichloromethane). ^{1}H-NMR (600 MHz, pyridine-d$_5$): δ = 8.96 (d, *J* = 8.0) and 8.91 (d, 1 H, *J* = 7.6), 8.47 (br s) and 8.40 (br s, 1 H), 8.37-8.33 (m, 1 H), 4.86-4.80 (m, 1 H), 3.90-3.87 (m) and 3.76-3.73 (m, 1 H), 3.41-3.32 (m, 2 H), 3.18-3.14 (m, 2 H), 2.78-2.66 (m, 2 H), 2.55-2.49 (m, 1 H), 2.31-2.23 (m, 2 H), 1.79-1.72 (m, 1 H), 1.69-1.62 (m, 2 H), 1.11 (t, *J* = 7.2) and 1.06 (t, 3 H, *J* = 7.2); ^{13}C-NMR (150 MHz, pyridine-d$_5$): δ =175.9, 173.6, 172.0, 55.8, 50.7, 42.0, 34.5, 33.9, 32.9, 28.9, 22.2, 15.5 ppm; $[\alpha]_D^{25}$ = +15.3 (c 0.3, methanol); HRMS (ESI+): calcd. for $C_{12}H_{23}N_4O_3$ [M + H$^+$]: 271.1765; found 271.1762.

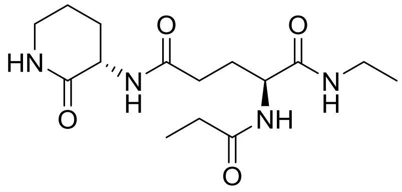

(*S*)-*N*[1]-Ethyl-*N*[5]-((*S*)-2-oxopiperidin-3-yl)-2-propionamidopentanediamide, glorinamide (**2**)

A mixture of amine **9b** (5 mg, 0.02 mmol, 1 equiv), propionic anhydride (24 mg, 0.20 mmol, 10 equiv), dimethylaminopyridine (1 mg, 20% w/w), diisopropylethylamine (25 mg, 0.20 mmol, 10 equiv) in dichloromethane (5 mL) was stirred at rt for 3 h. The reaction mixture was conc. in vacuo, and purified by flash column chromatography on silica gel (5% methanol in dichloromethane) to afford glorinamide **2** as a white solid. (6 mg, 0.02 mol, 97%). R_f = 0.1 (5% methanol in dichloromethane). ^{1}H-NMR (600 MHz, DMSO-d_6): δ = 8.00 (d, 1 H, *J* = 8.2), 7.88-7.84 (m, 2 H), 7.58 (br s, 1 H), 4.18-4.12 (m, 2 H), 3.14-3.10 (m, 2 H), 3.10-3.04 (m, 2 H), 2.17-2.03 (m, 4 H), 1.95-1.89 (m, 1 H), 1.87-1.67 (m, 4 H), 1.61-1.54 (m, 1 H), 1.02-0.97 (m, 6 H). ^{13}C-NMR (150 MHz, DMSO-d_6): δ = 173.3, 171.6, 171.5, 170.4, 52.5, 49.2, 41.5, 33.8, 32.3, 28.8, 28.7, 28.2, 21.5, 15.2, 10.3 ppm; $[\alpha]_D^{25}$ = –6.0 (c 0.2, methanol); HRMS (ESI+): calcd. for $C_{15}H_{27}N_4O_4$ [M + H$^+$]: 327.2027; found 327.2027.

References:

1. Abe, M.; Akiyama, T.; Umezawa, Y.; Yamamoto, K.; Nagai, M.; Yamazaki, H.; Ichikawa, Y.; Muraoka, Y. *J. Bioorg. Med. Chem.* **2005**, *13*, 785-797

2. Silva, L. L.; Joussef, A. C. *J. Nat. Prod.* **2011**, *74*, 1531–1534.

3. Malachowski, W. P.; Coward, J. K. *J. Org. Chem.* **1994**, *59*, 7625-7634

4. Ball, J. B.; Craik, D. J.; Alewood, P. F.; Morrison, S.; Andrews, P. R.; Nicholls, I. A. *Aust. J. Chem.* **1989**, *42,* 2171–2180.

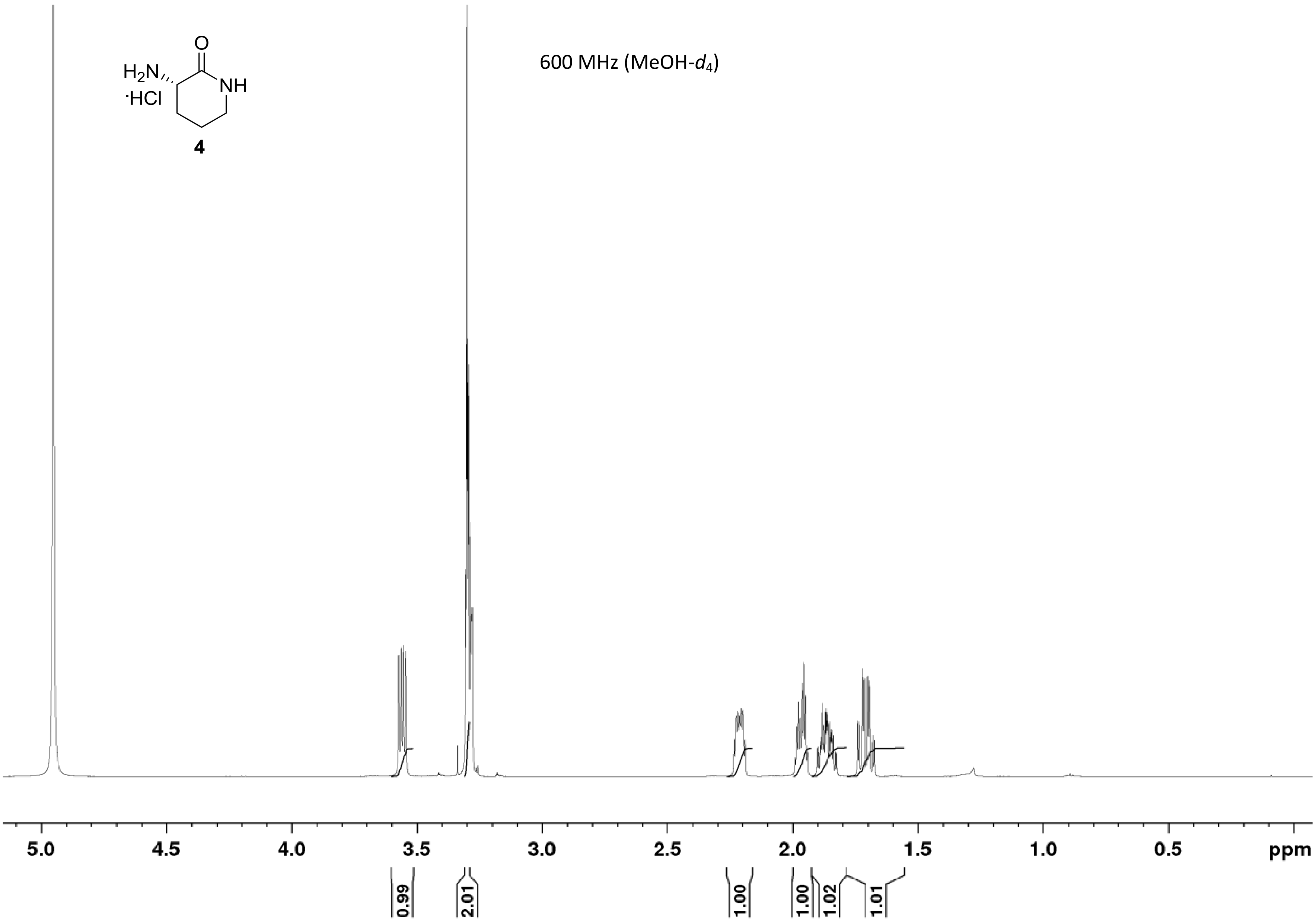

H2N
·HCl
O
NH
4
600 MHz (MeOH-d4)
5.0
4.5
4.0
3.5
3.0
2.5
2.0
1.5
1.0
0.5
ppm
0.99
2.01
1.00
1.00
1.02
1.01

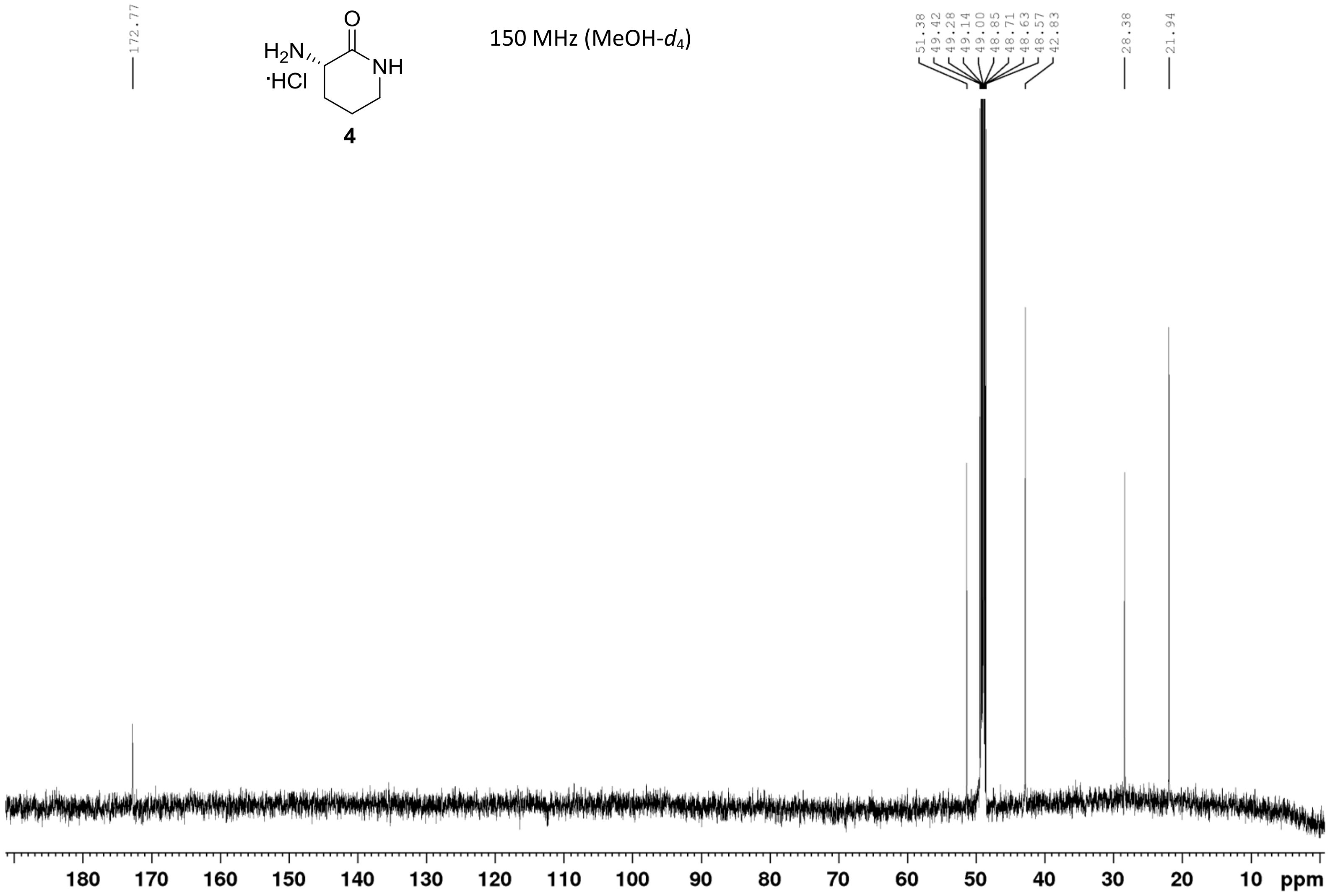

H2N
·HCl
NH
O
4
150 MHz (MeOH-d4)
172.77
51.38
49.42
49.28
49.14
49.00
48.85
48.71
48.63
48.57
42.83
28.38
21.94
180
170
160
150
140
130
120
110
100
90
80
70
60
50
40
30
20
10
ppm

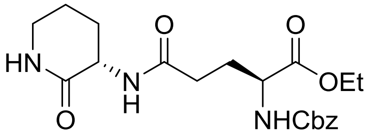

8a

300 MHz ($CDCl_3$)

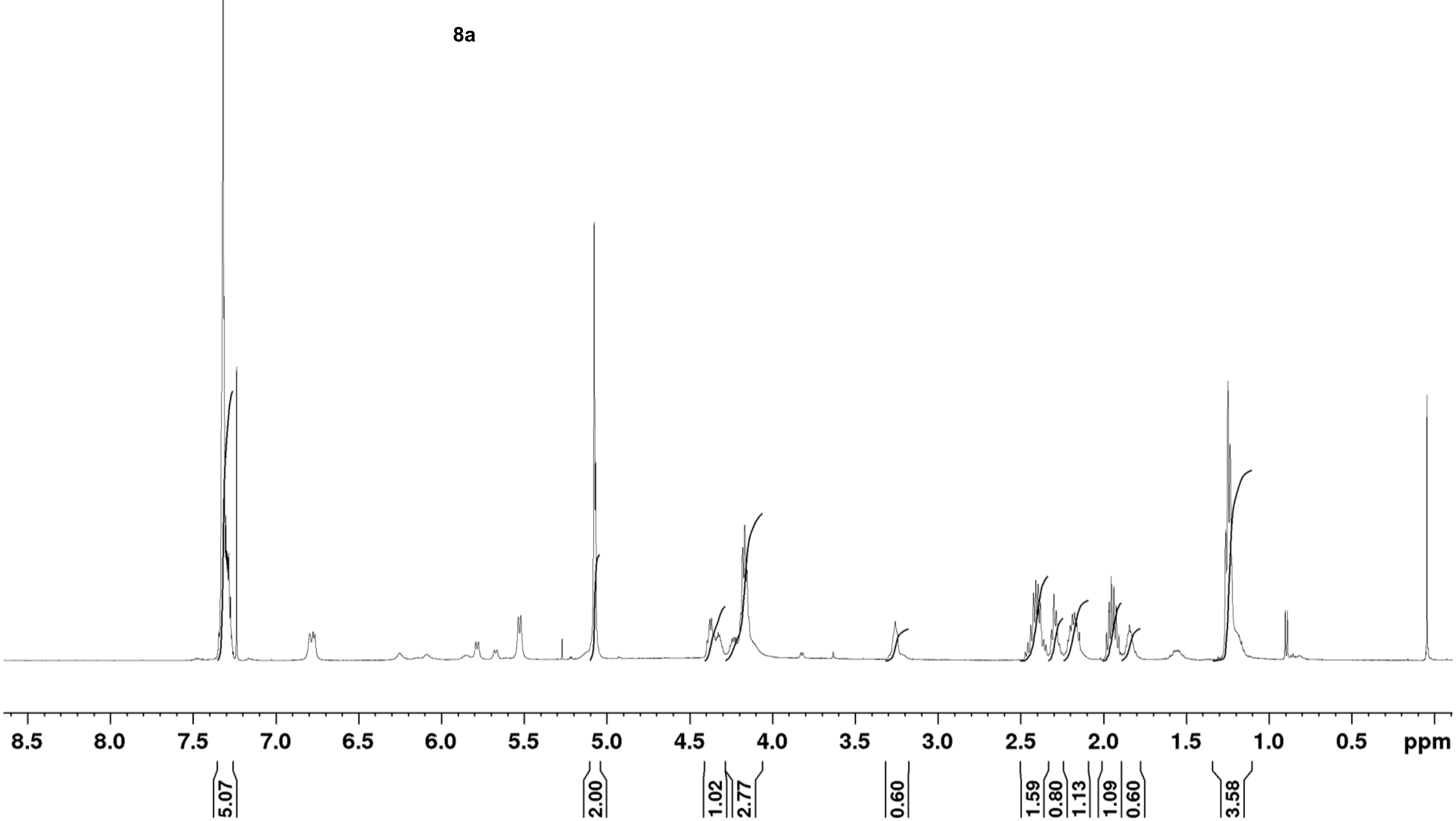

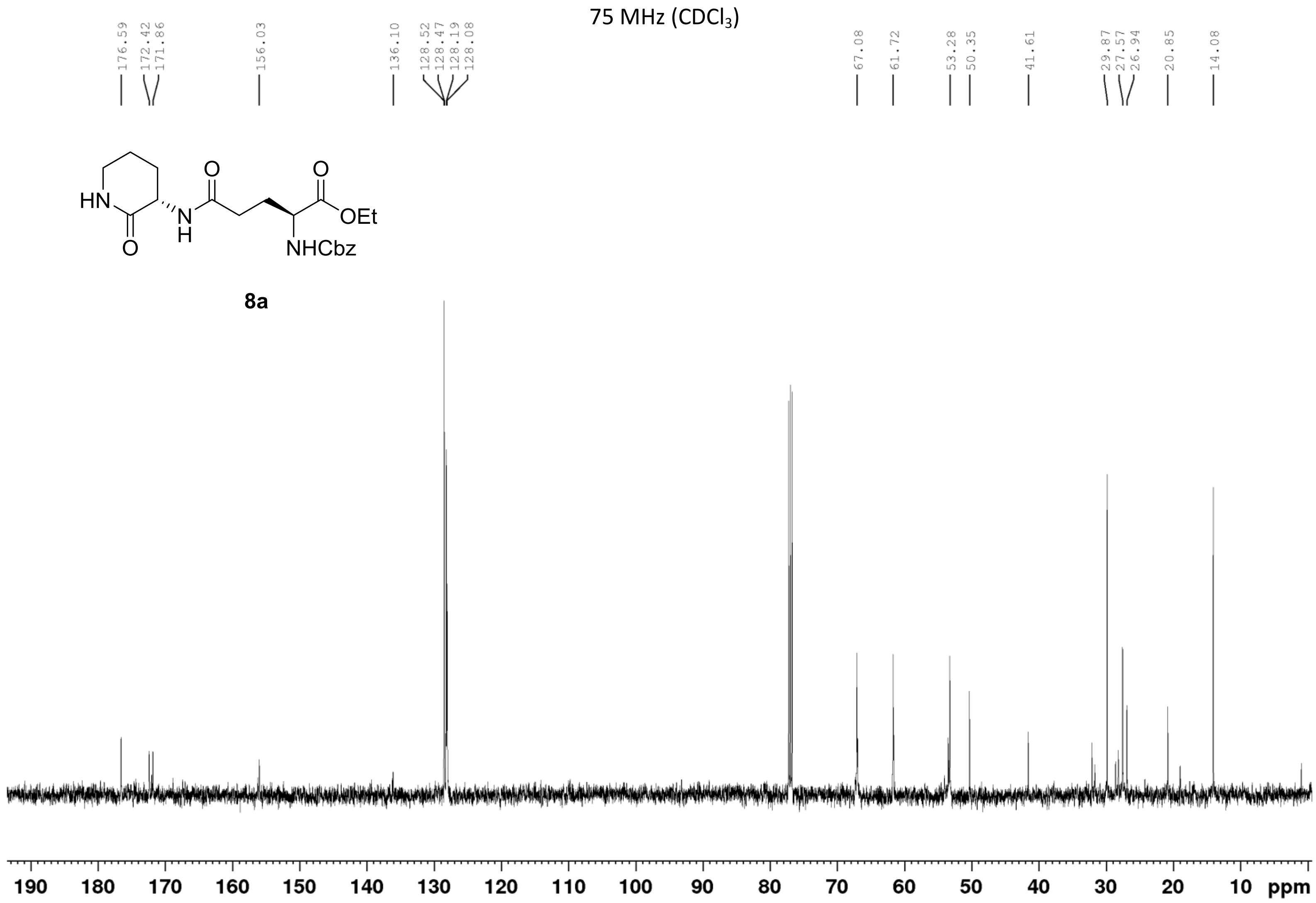
75 MHz (CDCl3)
176.59
172.42
171.86
156.03
136.10
128.52
128.47
128.19
128.08
67.08
61.72
53.28
50.35
41.61
29.87
27.57
26.94
20.85
14.08
HN
O
N
H
O
O
OEt
NHCbz
8a
190 180 170 160 150 140 130 120 110 100 90 80 70 60 50 40 30 20 10 ppm

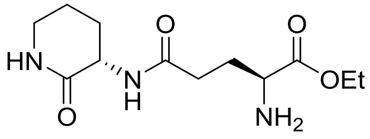

9a

500 MHz ($CDCl_3$)

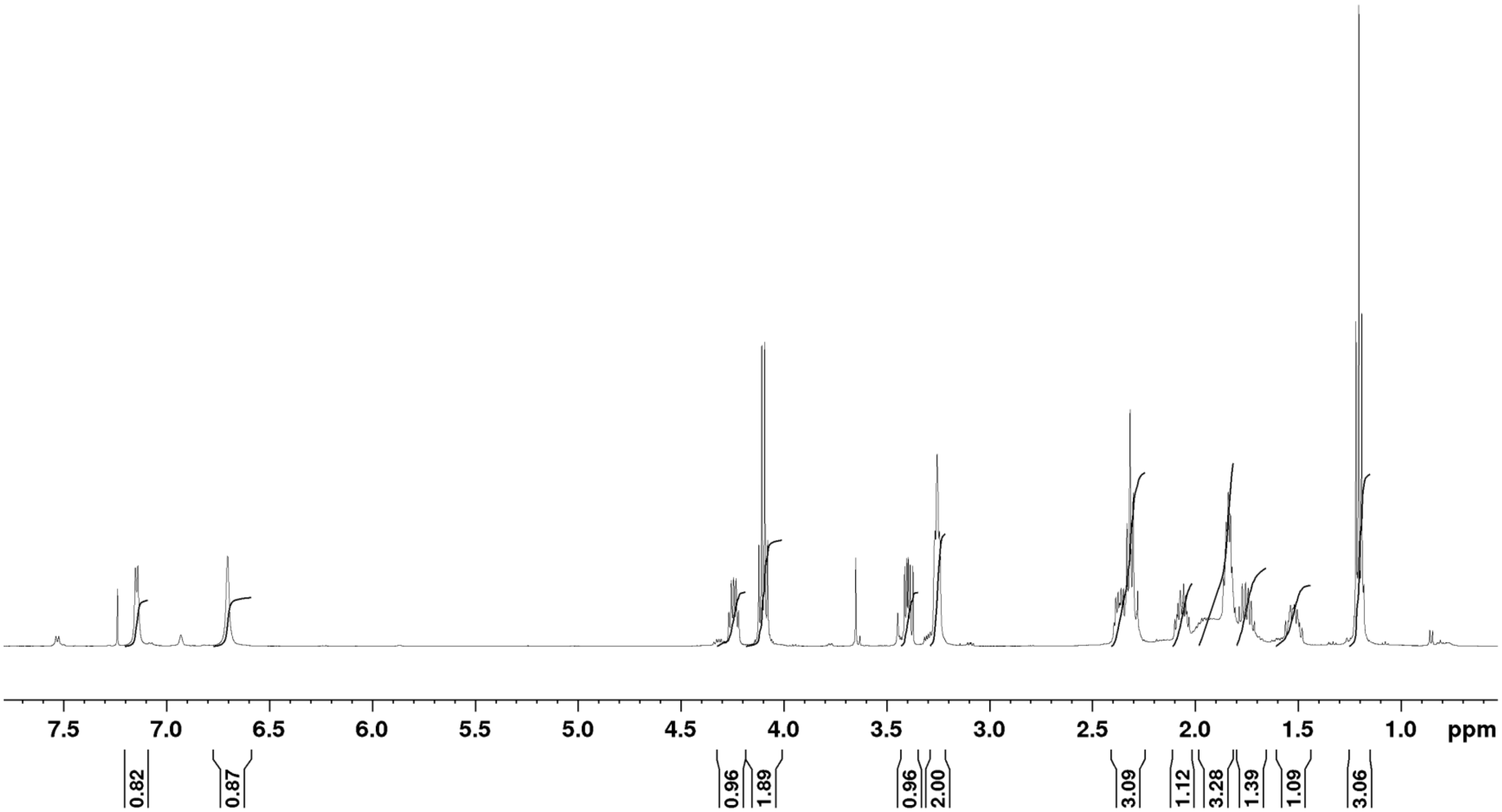

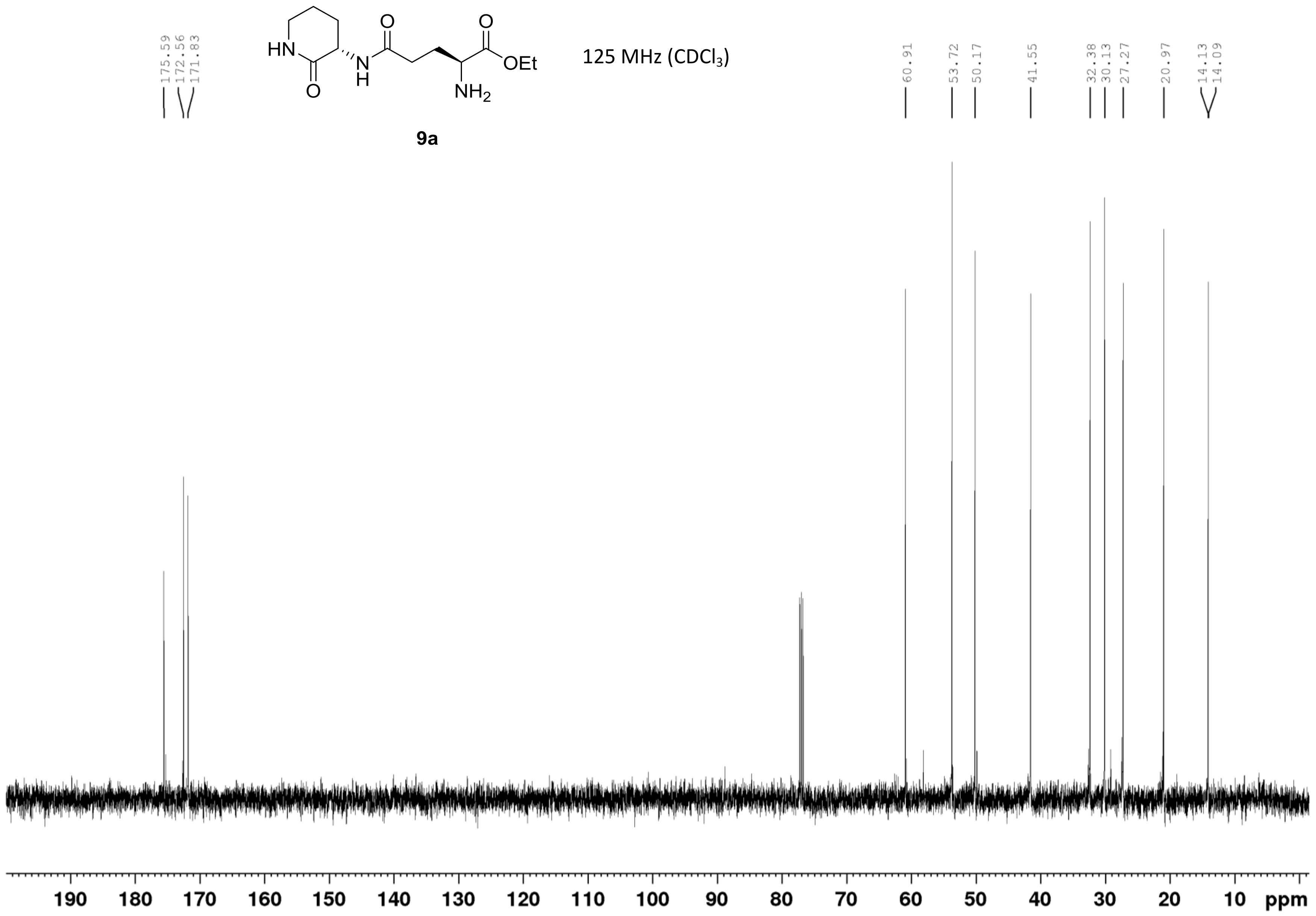
175.59
172.56
171.83
HN
O
N
H
O
O
OEt
NH2
9a
125 MHz (CDCl3)
60.91
53.72
50.17
41.55
32.38
30.13
27.27
20.97
14.13
14.09
190
180
170
160
150
140
130
120
110
100
90
80
70
60
50
40
30
20
10
ppm

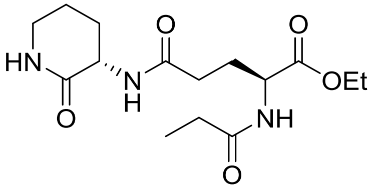

1

300 MHz ($CDCl_3$)

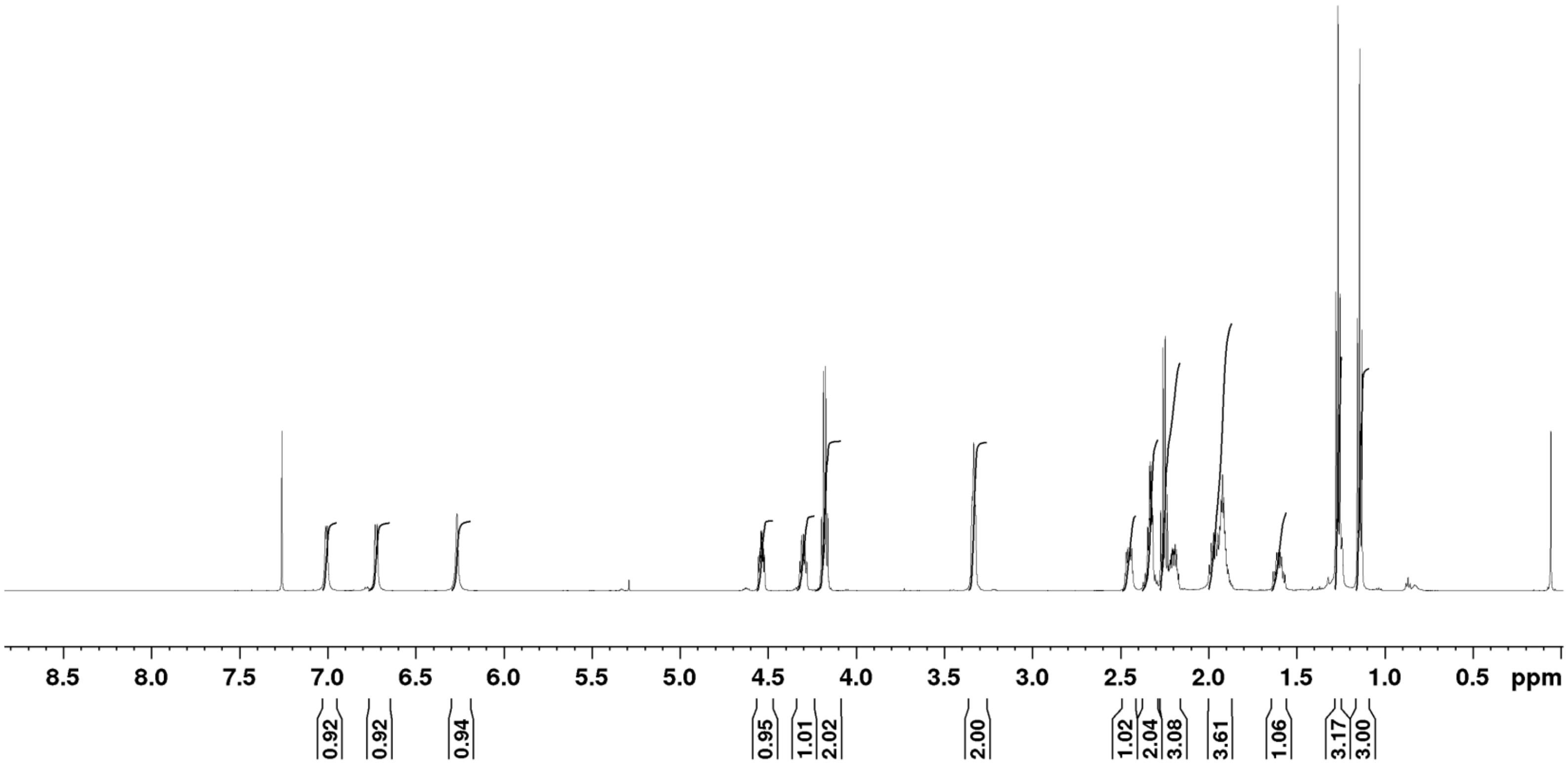

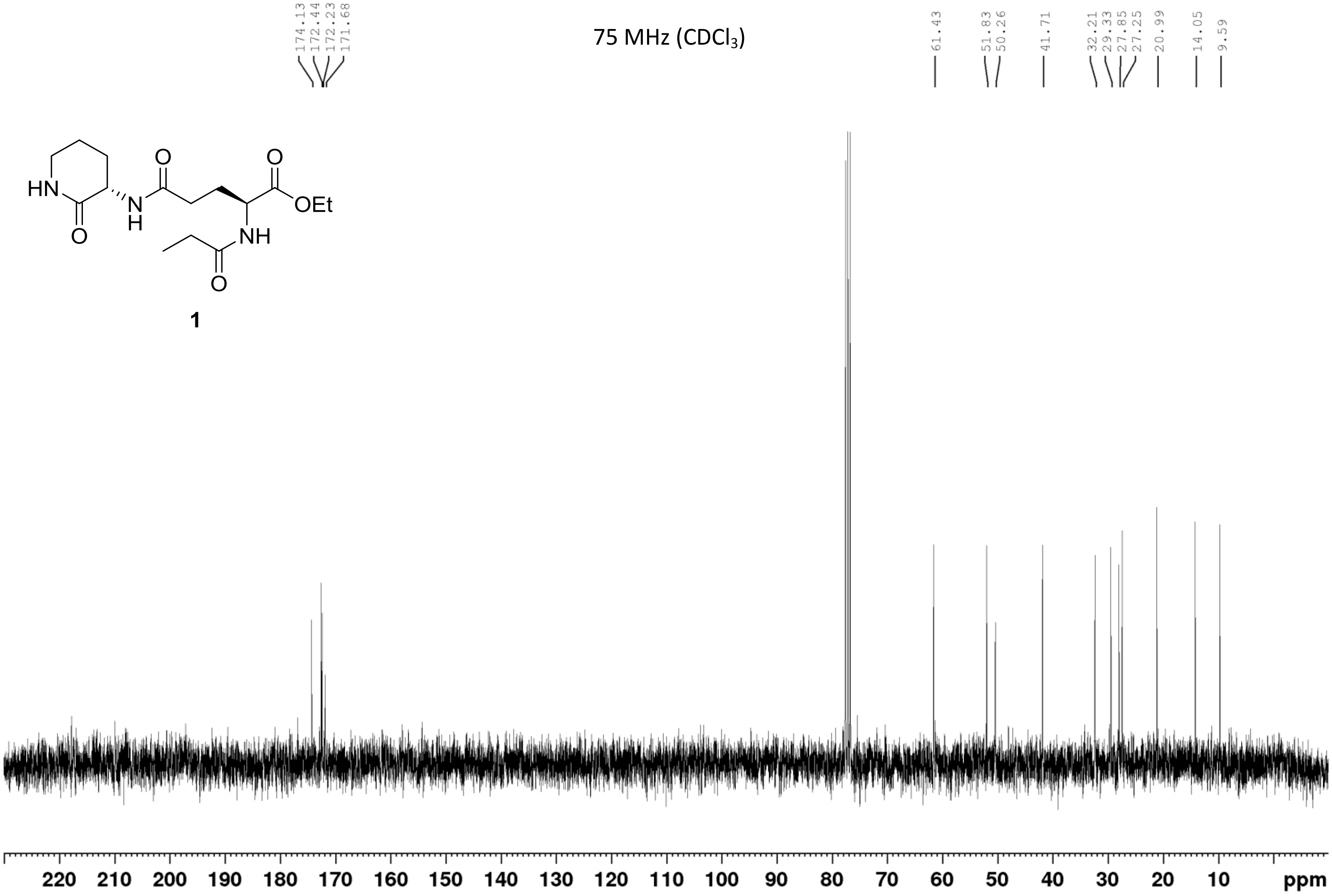

75 MHz ($CDCl_3$)
174.13
172.44
172.23
171.68
61.43
51.83
50.26
41.71
32.21
29.33
27.85
27.25
20.99
14.05
9.59
HN
O
N
H
O
O
OEt
NH
O
1
220 210 200 190 180 170 160 150 140 130 120 110 100 90 80 70 60 50 40 30 20 10 ppm

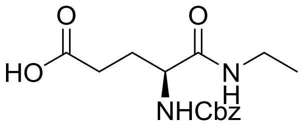

7b

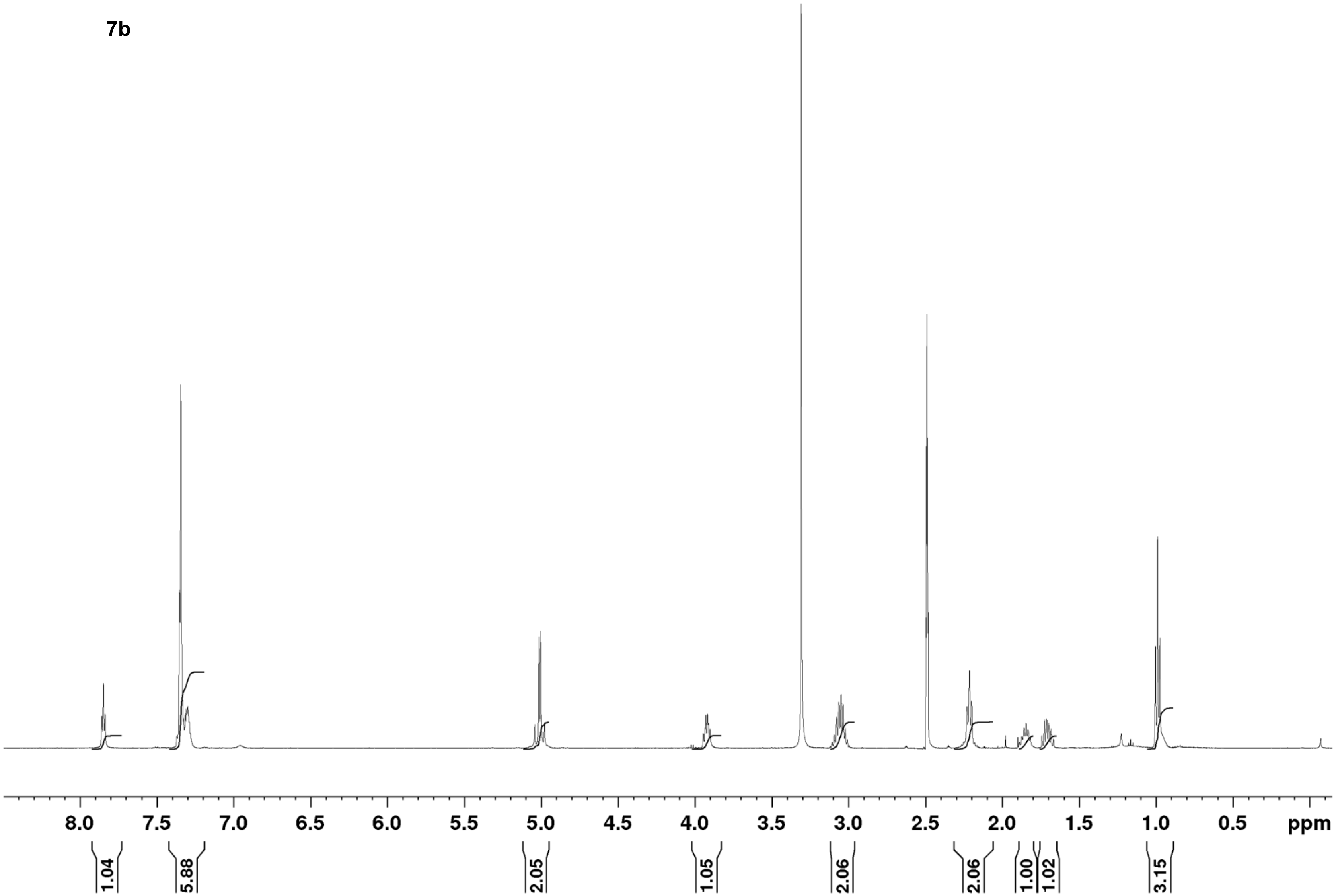
500 MHz (DMSO-d_6)
8.0
7.5
7.0
6.5
6.0
5.5
5.0
4.5
4.0
3.5
3.0
2.5
2.0
1.5
1.0
0.5
ppm
1.04
5.88
2.05
1.05
2.06
2.06
1.00
1.02
3.15

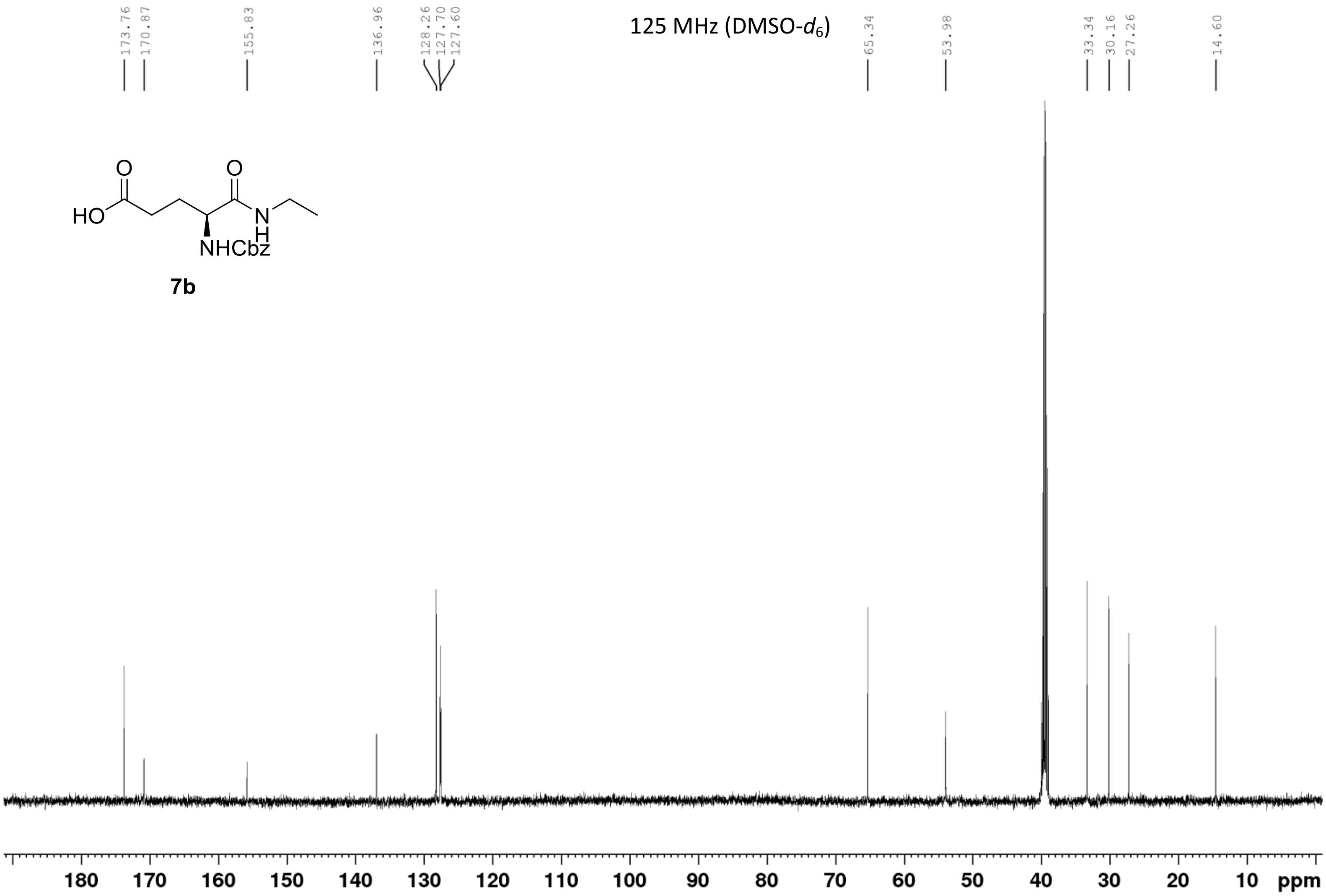
125 MHz (DMSO-d_6)
173.76
170.87
155.83
136.96
128.26
127.70
127.60
65.34
53.98
33.34
30.16
27.26
14.60
HO
O
O
N
H
NHCbz
7b
180
170
160
150
140
130
120
110
100
90
80
70
60
50
40
30
20
10
ppm

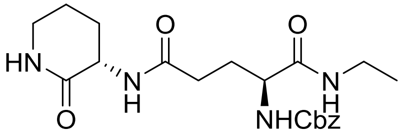

8b

500 MHz (DMSO-d_6)

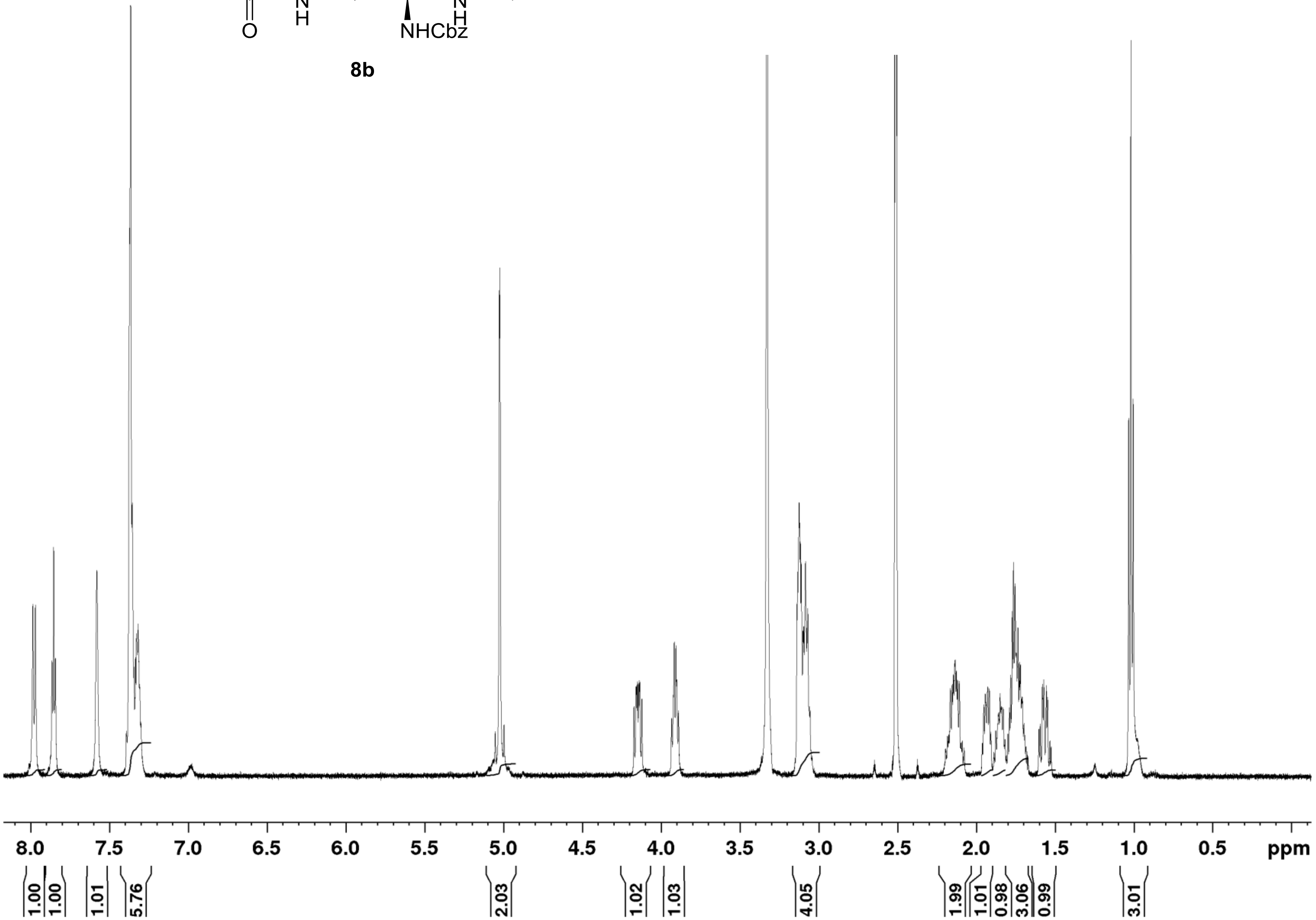

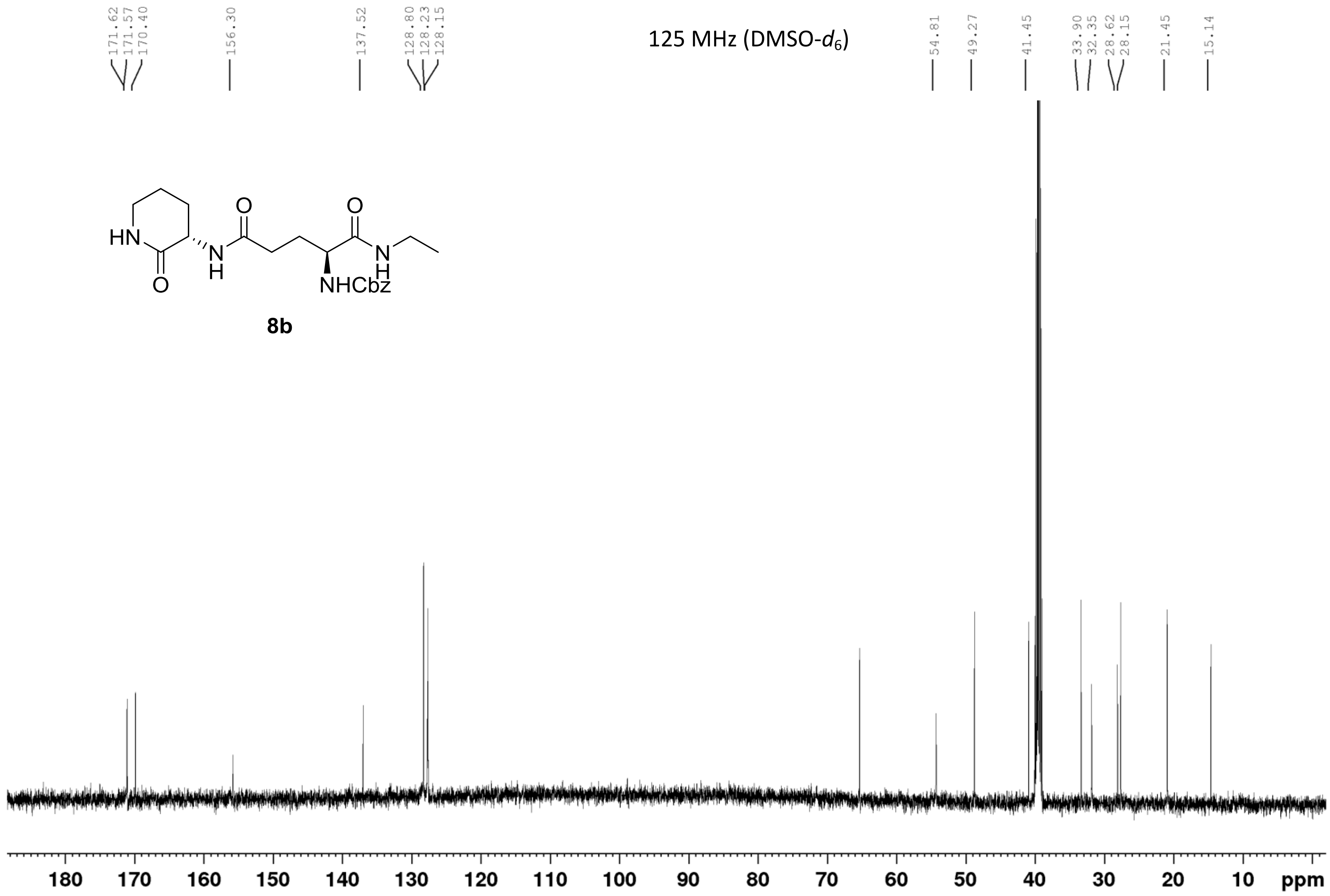
171.62
171.57
170.40
156.30
137.52
128.80
128.23
128.15
125 MHz (DMSO-d_6)
54.81
49.27
41.45
33.90
32.35
28.62
28.15
21.45
15.14
HN
O
N
H
O
O
N
H
NHCbz
8b
180 170 160 150 140 130 120 110 100 90 80 70 60 50 40 30 20 10 ppm

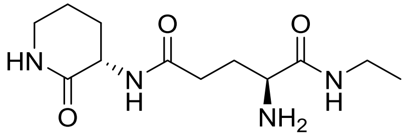

9b

600 MHz (Pyridine-d_5)

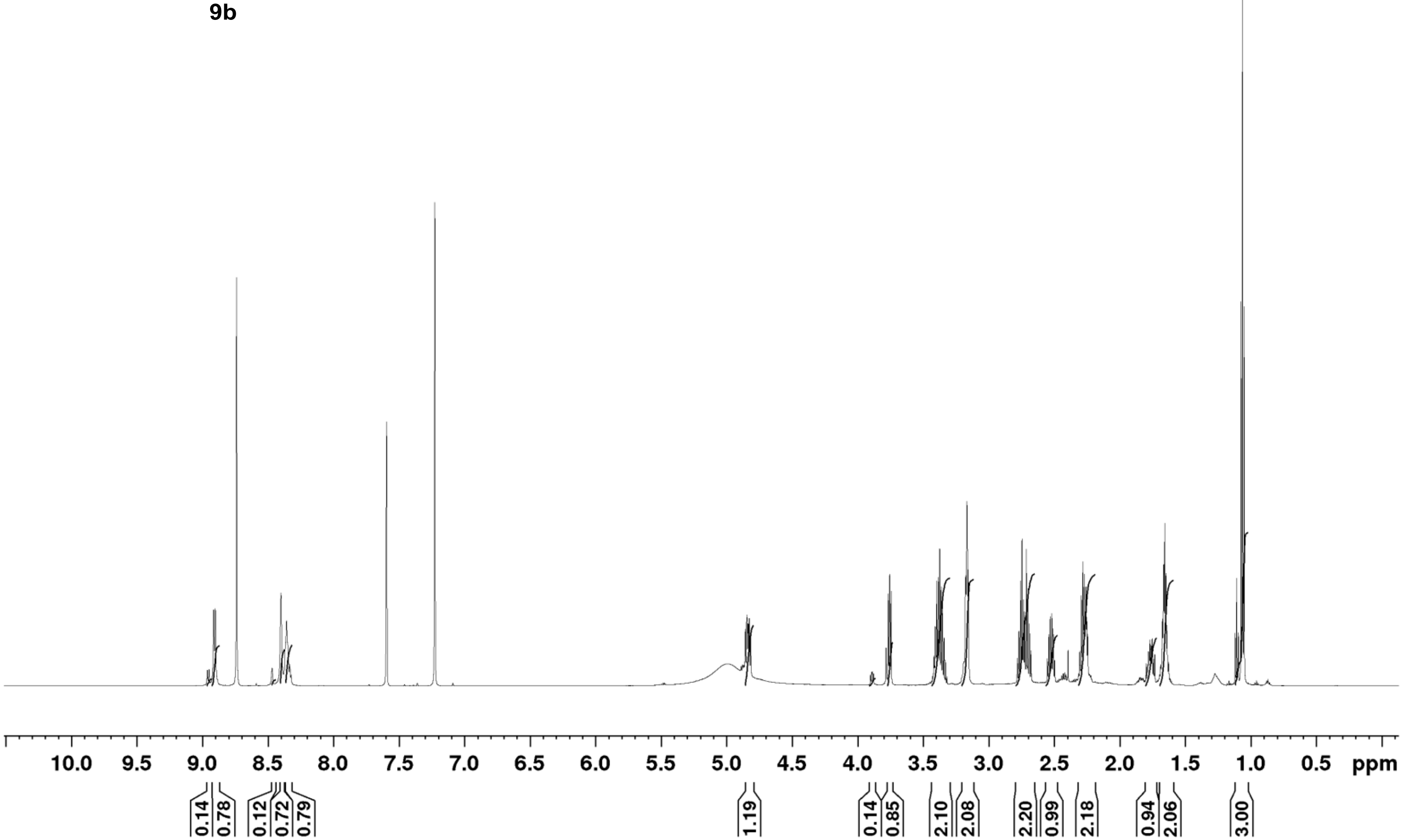

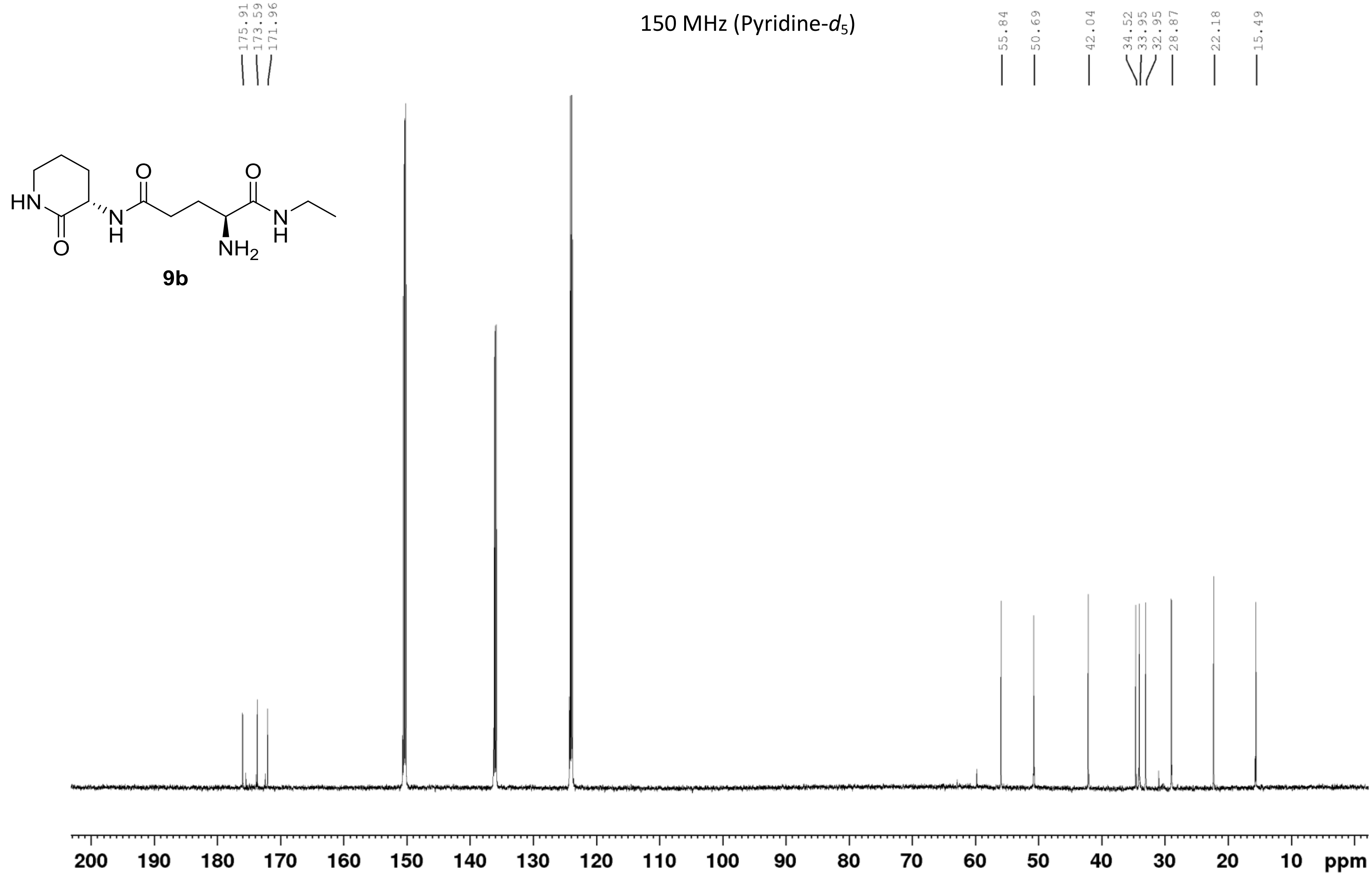

150 MHz (Pyridine-d_5)
175.91
173.59
171.96
55.84
50.69
42.04
34.52
33.95
32.95
28.87
22.18
15.49
HN
O
N
H
O
NH2
O
N
H
9b
200 190 180 170 160 150 140 130 120 110 100 90 80 70 60 50 40 30 20 10 ppm

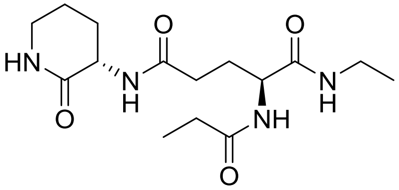

2

600 MHz (DMSO-d_6)

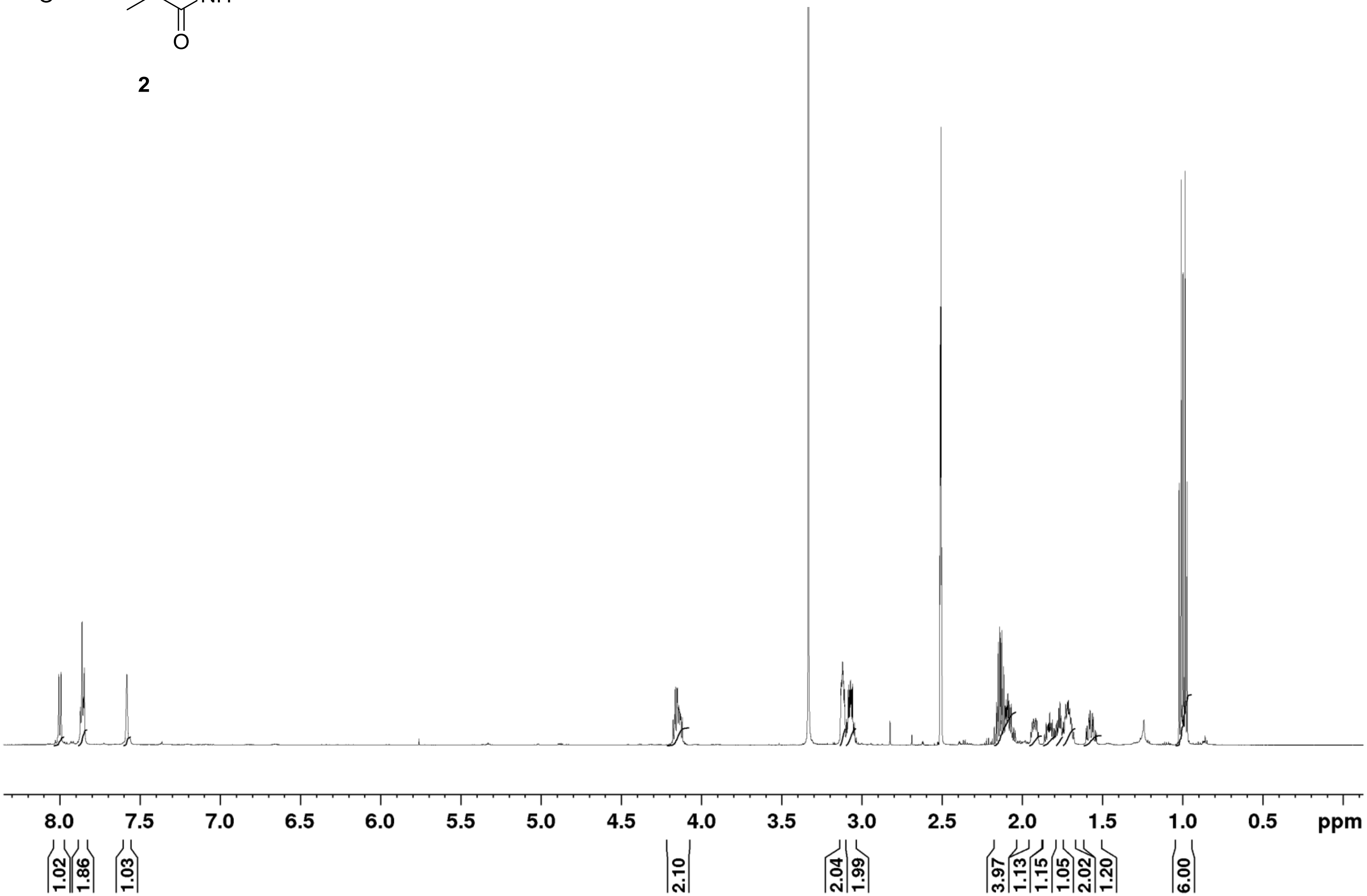

150 MHz (DMSO-d_6)

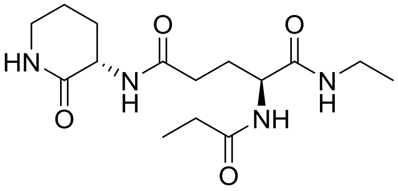

2

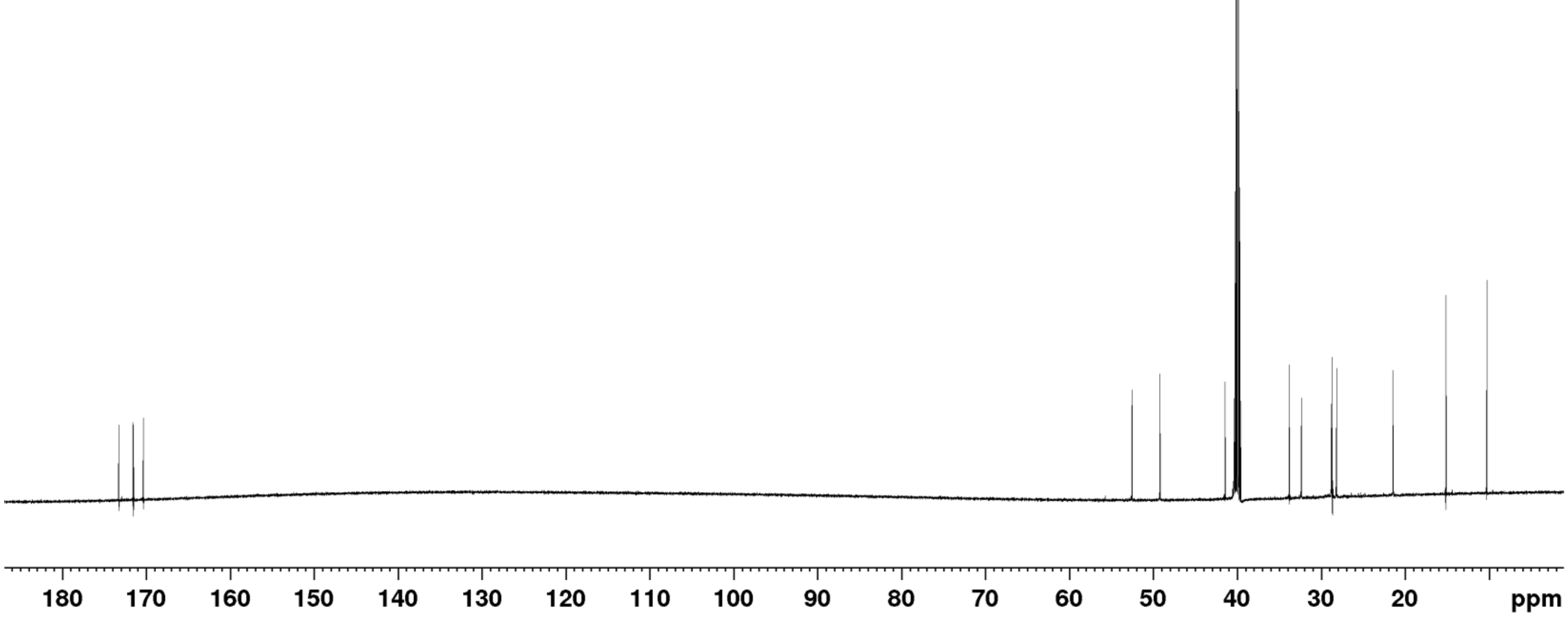

Supplement: File 1 — Detailed experimental procedures, compound characterization data, and copies of NMR spectra. [file Beilstein_J_Org_Chem-13-247-s001.pdf]
